# Long-Term Success of Regenerative Endodontic Treatment in Managing Traumatized Teeth: A Case Report With Seven-Year Follow-Up

**DOI:** 10.7759/cureus.57012

**Published:** 2024-03-27

**Authors:** Saeed Asgary

**Affiliations:** 1 Iranian Center for Endodontic Research, Research Institute of Dental Sciences, Shahid Beheshti University of Medical Sciences, Tehran, IRN

**Keywords:** regenerative endodontic therapy, clinical dentistry, tooth discoloration, calcium-enriched mixture cement, traumatized teeth

## Abstract

Traumatic injuries to maxillary incisors often result in complex dental complications, such as pulp necrosis and periapical pathology, particularly in young patients. Traditional root canal treatments may prove insufficient, especially for immature teeth requiring apexification. Regenerative endodontic treatment (RET) presents a promising alternative, aiming to eliminate infection while fostering root development and tooth vitality. This case report illustrates the successful management of a necrotic-infected traumatized maxillary incisor in a seven-year-old girl using RET. The treatment involved a meticulously planned protocol comprising disinfection, induction of bleeding, and placement of a calcium-enriched mixture (CEM) cement plug, followed by composite restoration. Remarkably, despite the initial detection of an endodontic lesion in the postoperative radiograph, the clinical outcomes remained aesthetically pleasing, with subsequent radiographs revealing regression of the apical lesion and complete tooth maturation over the seven-year follow-up period. This case highlights the efficacy and feasibility of RET using CEM in managing infected, traumatized teeth, emphasizing its potential for long-term healing and functional restoration. The absence of tooth discoloration further underscores the benefits of utilizing specific materials and protocols.

## Introduction

Traumatic dental injuries, particularly those affecting the maxillary incisors in pediatric patients, present significant challenges in clinical management [1]. When left untreated, these injuries can lead to a series of complications, starting with pulp necrosis and progressing to apical periodontitis. Traditional treatment modalities such as apexification and conventional root canal therapy may not always yield satisfactory outcomes, especially in cases involving immature teeth with open apices [2]. In response to these challenges, regenerative endodontic treatment (RET) has emerged as a promising alternative approach in clinical practice.

RET represents a paradigm shift in endodontic therapy, focusing on the regeneration of damaged dental tissues to restore function and vitality while concurrently eliminating infection [3]. This treatment modality capitalizes on the regenerative potential of dental pulp stem cells and growth factors present within the periapical tissues. By creating a conducive environment within the root canal system and utilizing biocompatible materials, RET aims to promote root development and preserve natural dentition [4].

Among the materials employed in RET, calcium-enriched mixture (CEM) cement has garnered attention for its favorable properties, including biocompatibility, antimicrobial activity, and ability to stimulate tissue regeneration. The use of CEM cement in RET has shown promising results in various clinical scenarios, including traumatized teeth with pulp necrosis and apical periodontitis [5-8].

Against this backdrop, this study endeavors to investigate the efficacy of RET utilizing CEM cement in managing traumatized teeth with pulp necrosis and apical periodontitis. Through a comprehensive evaluation of clinical and radiographic outcomes over a seven-year follow-up period, this study aims to provide insights into the long-term success and viability of RET as a conservative yet effective treatment approach. Additionally, it seeks to contribute to the expanding body of evidence supporting the utilization of CEM cement in regenerative endodontics, further advancing the field and enhancing treatment outcomes for patients with traumatic dental injuries.

## Case presentation

A seven-year-old female presented to the dental clinic with a traumatic injury to her left maxillary central incisor sustained approximately one month prior. The patient reported experiencing mild discomfort and occasional swelling in the affected tooth. Upon clinical examination, the tooth exhibited sensitivity to percussion and palpation, and the pulp space initially appeared filled with food impaction, which was subsequently removed, leaving the space empty. The adjacent teeth appeared normal, with no signs of mobility or sensitivity.

**Figure 1:**
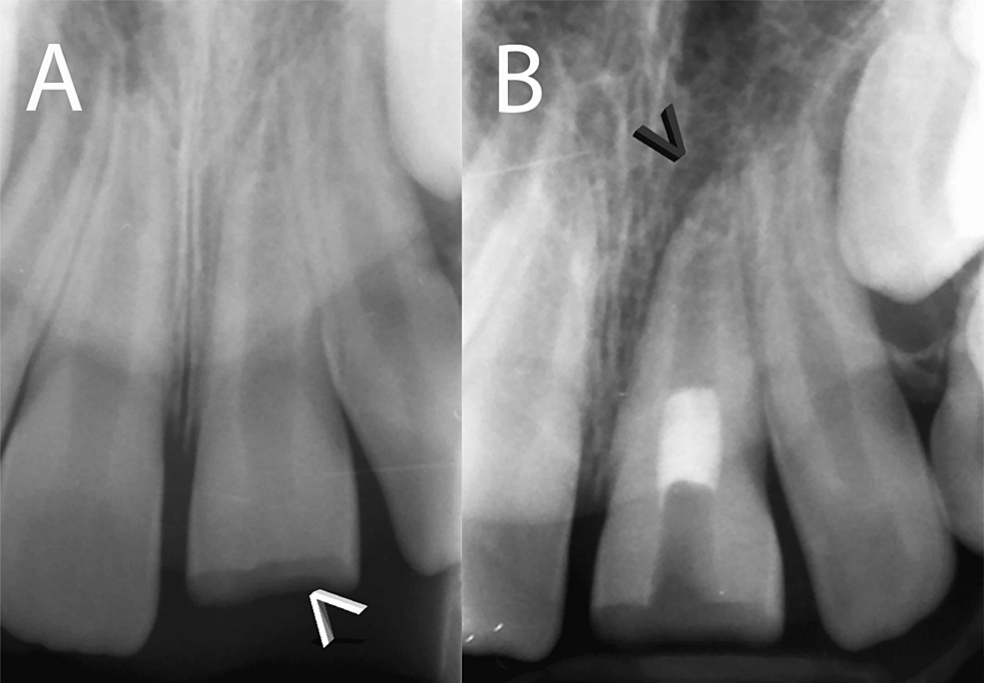
Initial presentation of the traumatized maxillary central incisor (A) Periapical radiograph taken immediately after trauma showing a fractured crown (indicated by the white arrowhead), an immature apex, and a normal PDL around the affected tooth. (B) Immediate postoperative periapical radiograph displaying the proper placement of the CEM plug in the midroot of the treated tooth. It is noteworthy that a large endodontic lesion is clearly visible (indicated by the black arrowhead). CEM: calcium-enriched mixture; PDL: periodontal ligament

A periapical radiograph taken immediately post-trauma revealed an immature apex and normal periodontal ligament (PDL) around the traumatized tooth (Figure 1A). Based on these clinical and radiographic findings, a diagnosis of pulp necrosis with an open apex was established for the involved tooth. The patient's medical and dental history was non-contributory, and parental consent was obtained for treatment.

After a thorough discussion of treatment options, including apexification, one-step apical plug, and RET, the decision was made to pursue RET due to its potential for promoting root development and preserving tooth vitality. Treatment commenced with a one-month application of a combination of modified triple antibiotics (penicillin G, metronidazole, and ciprofloxacin) [9] within the root canal space during the first session.

In the subsequent session, the root canal was gently cleaned and shaped using sodium hypochlorite, followed by copious irrigation with normal saline to remove the antibiotic paste. Subsequently, ethylenediaminetetraacetic acid (EDTA) was employed to prepare the root canal dentinal walls. Bleeding was induced, and CEM cement (BioniqueDent, Tehran, Iran) was carefully placed as a biomaterial plug over the newly formed blood clot (Figure 1B). A prefabricated fiber post was then inserted into the root canal, followed by the filling of light-cured resin-bonded dental composite restorative material to restore the tooth.

**Figure 2:**
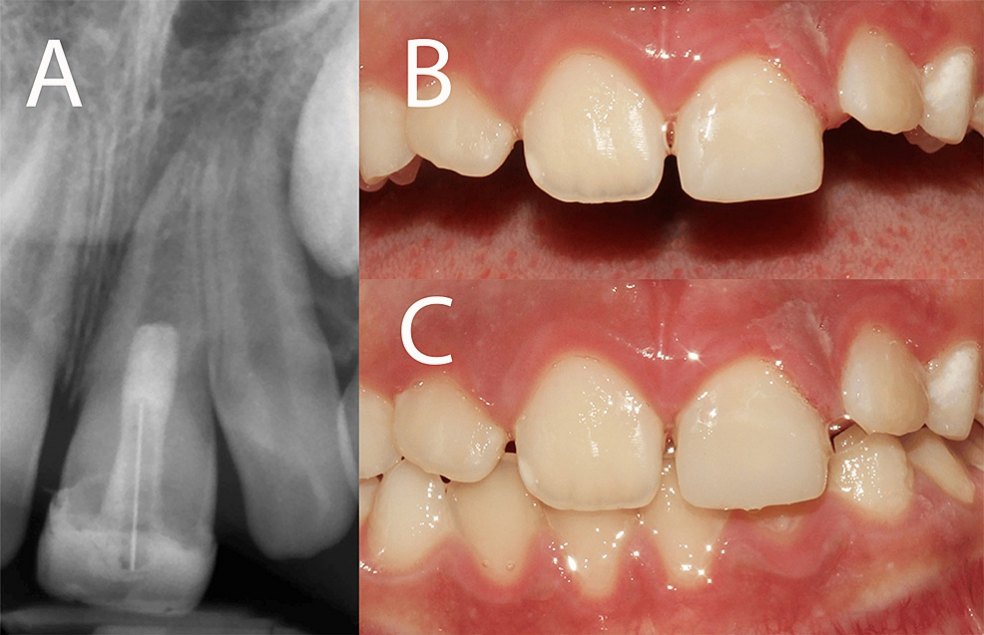
Completion of RET procedures and coronal restoration (A) Postperative periapical radiograph showing the successful placement of the CEM cement plug and a fiber post, along with a composite restoration. (B and C) Clinical images with open and closed occlusion taken immediately after treatment demonstrate favorable aesthetic outcomes. CEM: calcium-enriched mixture; RET: regenerative endodontic treatment

A postoperative radiograph revealed successful filling/sealing of the CEM cement plug and fiber post, with evidence of an endodontic lesion (Figure 2A). Despite this finding, the clinical outcomes from an aesthetic standpoint were deemed successful immediately after treatment (Figure 2B).

**Figure 3:**
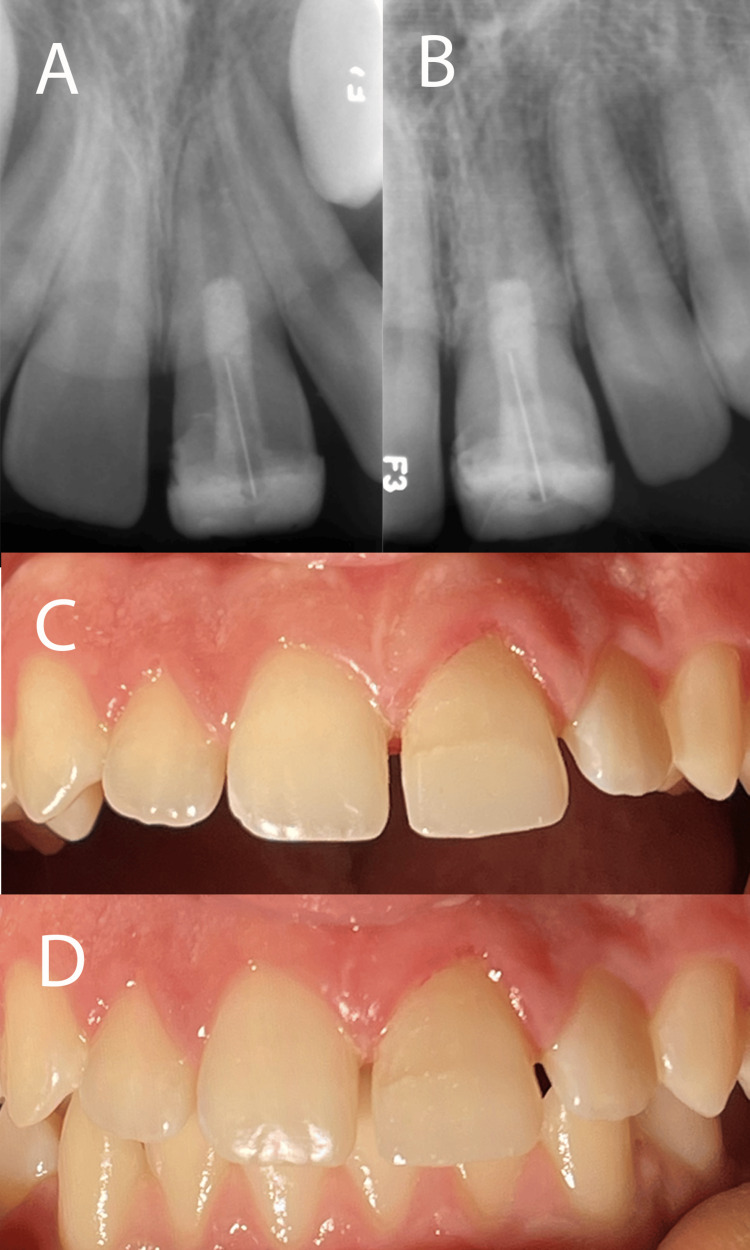
Short- and long-term follow-up outcomes (A) Periapical radiograph at the six-month follow-up, showing regression of the apical lesion. (B) Periapical radiograph at the seven-year follow-up, displayed complete root maturation alongside the normal PDL. (C and D) Clinical images with open and closed occlusion at the seven-year follow-up, demonstrating the absence of tooth discoloration, attributed to the use of triple antibiotics with penicillin and CEM cement. PDL: periodontal ligament; CEM: calcium-enriched mixture

At the six-month follow-up visit, radiographs demonstrated regression of the apical lesion (Figure 3A). Subsequent follow-up after seven years, when the patient was 14 years old, revealed complete tooth maturation, with the tooth appearing normal in the arch and functionally intact (Figure 3B). Notably, there was no evidence of tooth discoloration throughout the follow-up period, attributed to the use of modified triple antibiotics with penicillin and CEM cement, which is known to prevent discoloration (Figure 3C).

## Discussion

The management of traumatized teeth with pulp necrosis and periapical pathology presents challenges, particularly in young patients where traditional treatment modalities may be less effective. In this case, RET utilizing CEM cement was successfully employed to address these challenges. The decision to pursue RET was based on its potential to promote root development and preserve tooth vitality while eliminating infection. This treatment approach aligns with current trends in endodontics [4], emphasizing the importance of tissue regeneration and the preservation of natural dentition.

The utilization of a modified triple antibiotic paste as an intracanal medicament before inducing bleeding and applying CEM cement is noteworthy [9]. This tailored antibiotic regimen was intended to eradicate infection within the root canal system and establish an environment conducive to tissue regeneration. Despite ongoing debates regarding the use of antibiotics in endodontic therapy due to concerns such as tooth discoloration, antibiotic resistance, and cytotoxicity, their application, in this case, seemed to have contributed to successful outcomes without any discernible adverse effects, particularly tooth discoloration.

CEM cement, chosen as the biomaterial plug, offers several advantages in RET. It exhibits excellent biocompatibility, antimicrobial properties, and the ability to induce mineralization and tissue regeneration. Furthermore, CEM cement has been reported to have superior sealing ability and resistance to bacterial leakage compared to other endodontic materials. The absence of tooth discoloration observed in this case, despite long-term follow-up, underscores the importance of material selection in preventing aesthetic complications associated with endodontic treatment.

Radiographic findings demonstrated regression of the apical lesion over the follow-up period, indicating successful healing and resolution of periapical pathology. Additionally, complete tooth maturation with a normal PDL was observed, confirming the efficacy of RET in promoting root development in immature teeth with open apices. However, long-term follow-up studies with larger sample sizes are needed to validate the efficacy and predictability of RET in managing traumatized teeth.

## Conclusions

The positive results observed in this case highlight the potential of RET and its ability to improve endodontic therapy. By combining RET with CEM cement, damaged teeth with pulp necrosis and periapical pathology have shown significant improvement. This approach demonstrates promising results in preserving natural teeth and facilitating functional restoration, especially in younger patients. However, to comprehensively assess the long-term effectiveness and optimize the protocols of RET in endodontic practice, further research and prospective clinical trials are imperative.
